# Influence of Repressive Coping Style on Cortical Activation during Encoding of Angry Faces

**DOI:** 10.1371/journal.pone.0112398

**Published:** 2014-12-11

**Authors:** Astrid Veronika Rauch, Lena ter Horst, Victoria Gabriele Paul, Jochen Bauer, Udo Dannlowski, Carsten Konrad, Patricia Ohrmann, Harald Kugel, Boris Egloff, Volker Arolt, Thomas Suslow

**Affiliations:** 1 Department of Psychiatry, University of Muenster, Muenster, Germany; 2 Department of Psychiatry, University of Marburg, Marburg, Germany; 3 Department of Clinical Radiology, University of Muenster, Muenster, Germany; 4 Department of Psychology, University of Mainz, Mainz, Germany; 5 Department of Psychosomatic Medicine, University of Leipzig, Leipzig, Germany; University of California, San Francisco, United States of America

## Abstract

**Background:**

Coping plays an important role for emotion regulation in threatening situations. The model of coping modes designates repression and sensitization as two independent coping styles. Repression consists of strategies that shield the individual from arousal. Sensitization indicates increased analysis of the environment in order to reduce uncertainty. According to the discontinuity hypothesis, repressors are sensitive to threat in the early stages of information processing. While repressors do not exhibit memory disturbances early on, they manifest weak memory for these stimuli later. This study investigates the discontinuity hypothesis using functional magnetic resonance imaging (fMRI).

**Methods:**

Healthy volunteers (20 repressors and 20 sensitizers) were selected from a sample of 150 students on the basis of the Mainz Coping Inventory. During the fMRI experiment, subjects evaluated and memorized emotional and neutral faces. Subjects performed two sessions of face recognition: immediately after the fMRI session and three days later.

**Results:**

Repressors exhibited greater activation of frontal, parietal and temporal areas during encoding of angry faces compared to sensitizers. There were no differences in recognition of facial emotions between groups neither immediately after exposure nor after three days.

**Conclusions:**

The fMRI findings suggest that repressors manifest an enhanced neural processing of directly threatening facial expression which confirms the assumption of hyper-responsivity to threatening information in repression in an early processing stage. A discrepancy was observed between high neural activation in encoding-relevant brain areas in response to angry faces in repressors and no advantage in subsequent memory for these faces compared to sensitizers.

## Introduction

Coping is the process of attempting to manage the demands created by stressful events [1]. Coping strategies can be adaptive or maladaptive. The development of psychiatric diseases and the maintenance of physical health is influenced by coping, which can also influence interventions by aiming to reduce the health risks of stress [2]. Therefore, knowledge about coping is important for developing a better understanding of psychiatric diseases.

The model of coping modes [3], [4] describes habitual behavior tendencies in reaction to anxiety-provoking and threatening situations. Referring to this model, the terms ‘repression’ and ‘sensitization’ are introduced as independent coping styles. Repression is characterized by cognitive avoidance of threatening stimuli in order to shield the organism from distressing stimuli and to prevent an uncontrollable increase in arousal. The behavioral aim of repressors is to divert attention from threat-relevant cues. In contrast, sensitization is associated with vigilance and the ability to tolerate arousal but not uncertainty.

Although repressors describe themselves as low-anxious, they exhibit higher than average levels of physiological arousal [5]–[7] and are at an increased risk of stress related health problems [8]. The use of avoidant coping strategies is associated with a worse outcome in depression and several somatic diseases (for a review, see [2]).

Previous coping studies have revealed several differences in memory processing for emotional stimuli. Generally, repressors tend to avoid threats in order to prevent arousal, but the repressive discontinuity hypothesis postulates that, when exposed, repressors are not generally insensitive to threat at an early stage of memory processing. Instead, repressors showed a decline in memory for threatening stimuli after 3 days, but not during immediate recall (30 minutes after stimulus exposure) [9], [10]. However, the discontinuity hypothesis has not been tested in the context of neuroimaging. Thus, the cerebral correlates of encoding remain unclear in repression.

Only a few imaging studies have investigated emotion processing in repression. Sander et al. [11] found an increased activation in repressors in left temporoparietal regions during the presentation of sad intonations. Cortical activation was also found to be larger for repressors in prefrontal areas. In a previous study [12] we demonstrated that repressors showed heightened activation in frontal and temporo-occipital areas during the perception of fearful and happy faces. Repressors exhibited an increased connectivity between medial prefrontal areas and the amygdala during the presentation of fearful faces.

Facial expressions play an important role in social signaling and for determining environmental conditions [13], [14]. Correctly interpreting threatening faces is crucial for detecting environmental danger. So far, emotional faces have not been applied to test the discontinuity hypothesis. Hence, we employed emotional faces in this context. In the past, several functional magnetic resonance imaging (fMRI) studies investigated emotional face processing. Increased activation during the processing of emotional faces was found in several regions, including prefrontal, temporoparietal, limbic, visual areas, amygdala, putamen and cerebellum ((for reviews see [15]–[17]). A recent study [18] investigated memory for emotional stimuli and revealed several areas that were involved in recognizing emotional faces. These included prefrontal and frontal regions, as well as temporal and parietal areas. Encoding of emotional faces was associated with activity in the dorsolateral prefrontal cortex (DLPFC), frontal areas, amygdala, temporo-occipital regions and parietal lobule [19], [20].

Taken together, the neurobiological mechanisms of the repressive discontinuity hypothesis in the context of processing emotional faces remain unknown. Therefore, the aim of this fMRI study is to test this hypothesis at a neurobiological level. Repressors are expected to evaluate emotional faces as less threatening than sensitizers. Based on previous studies it is hypothesized that repressors will show an increased response to threatening faces (i.e. angry and fearful facial expression) in cortical regions which are known to play a role in emotion regulation and memory encoding, such as the prefrontal and temporo-parietal areas. Therefore, repressors' memory for threatening faces should be better directly after encoding compared to that of sensitizers. Furthermore, it was hypothesized that due to cognitive avoidance repressors will show a decreased memory for threatening faces after three days compared to sensitizers.

## Methods

### Participants

Forty healthy female, right-handed university students participated in this fMRI study. All subjects had no history of psychiatric, neurological, or other relevant medical diseases, were free of psychotropic medication and had a normal or corrected-to-normal (by contact lenses) vision. Handedness was assessed with the Handedness Questionnaire [21]. Visual acuity was checked prior the fMRI session by reading a miniature Snellen eye chart. Study participants were selected from a sample of 150 students on the basis of their scores on the German version of the Mainz Coping Inventory, MCI [22]. Twenty persons with high scores on the cognitive avoidance scale (>66th percentile of the screening sample) and low scores on the vigilance scale (<33th percentile) were included as consistent repressors in the present study, whereas 20 persons with high scores on the vigilance scale (>66th percentile of the screening sample) and low scores on the cognitive avoidance scale (<33th percentile) were included as consistent sensitizers. Cronbach's α was 0.84 for the MCI avoidance scale and 0.89 for the MCI vigilance scale. Repressors differed significantly (Ps<0.001) from sensitizers on both MCI scales (see table 1). Subjects were screened for imaging safety concerns, and gave written informed consent, which was approved by the ethics committee of the Medical Faculty at the University of Muenster. All subjects received a compensation of 35 EUR after their participation. The same subjects participated also in a second experiment which was published previously [23]. In this experiment a detection task was applied to measure automatic responsivity to facial emotions as a function of coping style. The detection task was always conducted before the present memory experiment and used different facial stimuli [24]. In the detection task participants were shown happy, angry, fearful, and neutral facial expressions masked by neutral faces and they had to guess which of these facial expressions has been briefly presented [23].

**Table 1:** Descriptive characteristics of study participants.

	Sensitizers		Repressors	
	*Mean*	*SD*	*Mean*	*SD*
MCI vigilance	28.2	3.3	14.0	2.7
MCI cognitive avoidance	16.5	4.1	27.5	2.4
Age	22.9	2.3	22.4	2.7
Verbal IQ (MWT-B)	115.8	10.9	117.0	13.0
Picture Completion (WAIS-R)	14.5	1.3	14.2	1.9
STAI-State	36.0	6.7	31.6	4.7
STAI-Trait	36.1	8.7	29.2	4.2
BDI	3.9	4.7	2.0	2.0

*Note.* MCI: Mainz Coping Inventory; WAIS-R: Wechsler Adult Intelligence Scale – revised form; STAI: State-Trait Anxiety Inventory, state and trait version; BDI: Beck Depression Inventory.

### Questionnaires and Neuropsychological Measures

The MCI [25] is a stimulus-response inventory that assesses vigilant and cognitive avoidant coping strategies in 4 ego-threatening (e.g., public speaking) and 4 physically threatening situations (e.g., riding with an inexperienced driver). For each situation, 5 vigilant or sensitizing items (e.g., information search, anticipation of negative events) and 5 cognitively avoidant or repressive items (e.g., denial, attention diversion) are administered in a true—false response format. Scored answers are summed for vigilant and avoidant items across all 8 situations.

The State-Trait Anxiety Inventory, STAI, [26] was administered in its German version [27], as well as the Beck Depression Inventory, BDI, [28], [29]. The Multiple choice vocabulary test, MWT-B, [30] was applied to assess the verbal intelligence of study participants. Picture Completion is a subtest of the Wechsler Adult Intelligence Scale, WAIS-R, [31] which measures visual perception and recognition of essential details of objects. Statistical analyses were performed using SPSS Statistics 17.0.0 (Statistical Package for the Social Sciences, 2008).

### fMRI Stimulus Material and Procedure

The study protocol included an event-related fMRI experiment and two sessions of face recognition tasks outside the scanner after the fMRI session. The first was 30 minutes after, and the second 3 days after. All three tasks included colored facial stimuli (angry, happy, fearful and neutral) [32] of 15 male and 15 female subjects. Facial stimuli were presented pseudo-randomly. Only during the fMRI experiment was a no face condition (a grey rectangle) added to optimize the paradigm with null events [33]. In the fMRI experiment, 150 trials were presented. Each emotional face condition and the no face condition consisted of 30 trials. The total duration of the experiment was 17.5 minutes. A fixation cross of 500 ms preceded the emotion face, which was presented for 3000 ms followed immediately by a time interval (3500 ms) for rating the facial emotion as rather threatening (−0.5, −1.5) or rather non-threatening (+0.5, +1.5) by pressing a button. Thus, both negative numbers were accompanied by the adjective “threatening”, whereas both positive numbers were accompanied by the adjective “non-threatening”. One-half of the samples gave positive responses with the left hand and the other half used the right hand for positive responses. Subjects were instructed to evaluate and memorize the faces. The experiment was programmed using the software package Inquisit [34]. Two fixed random orders were presented which were counterbalanced across subjects. The type of evaluative response and reaction latencies were registered. Face stimuli were presented via a projector (Sharp XG-PC10XE). The head position was stabilized with a vacuum head cushion.

The two recognition tasks consisted of 120 original faces shown during the fMRI session and 120 distractors (30 angry, 30 happy, 30 fearful and 30 neutral faces of other individuals [32]). Two fixed random orders of faces were presented. The order of faces was counterbalanced across subjects. Subjects were instructed to rate whether they have seen the faces during the fMRI experiment or not. Participants rated the facial emotion as original seen during the fMRI session (“Yes”) or as distractors (“No”) by pressing a button. Each stimulus was presented until the subject responded.

### fMRI Data Acquisition and fMRI Data Analyses

T_2_* functional data were acquired at a 3-Tesla scanner (Gyroscan Intera 3T, Philips Medical Systems, Best, The Netherlands) using a single shot echoplanar sequence with parameters selected to minimize distortion while retaining adequate signal to noise ratio and T_2_* sensitivity, according to suggestions made by Robinson et al. [35]. Volumes consisting of 36 axial slices were acquired (matrix 64^2^, resolution 3.5 mm * 3.5 mm * 3.5 mm; time repetition  = 2.5 s, time echo  = 35 ms, flip angle  = 90°) 420 times in an event-related design. T_1_-weighted inversion recovery and a high-resolution T_1_-weighted 3D sequence (isotropic pixel, 0.5^3^ mm) were also acquired. Functional imaging data were motion corrected using a set of 6 rigid body transformations determined for each image. We applied slice timing and images were spatially normalized to standard MNI space (Montreal Neurological Institute) as well as smoothed (Gaussian kernel, 6-mm FWHM) using Statistical Parametric Mapping 5 (SPM5; Wellcome Department of Cognitive Neurology, London, UK, http://www.fil.ion.ucl.ac.uk/spm) implemented in Matlab 7.1. Checks on motion artifacts were performed and analyses used the estimated motion parameters as covariates of no interest in the event related design matrix. Statistical analysis was performed by modeling the different conditions (angry, fearful, happy, and neutral) as variables within the context of the general linear model.

A whole-brain analysis was conducted to determine which brain regions were differentially activated as a function of coping style. For each emotion condition (fearful, angry, and happy), activation data (t maps) were calculated for subjects relative to the neutral control face condition. Furthermore, activation data of the neutral versus angry contrast were calculated for each subject. Random effects analysis (t-tests for independent samples) was performed to examine brain activation differences between groups (on the contrasts: fearful vs. neutral, angry vs. neutral, happy vs. neutral and neutral vs. angry). Coordinates of significant activations (p<0.05, k = 50, False Discovery Rate) were converted into Talairach and Tournoux [36] space using the Talairach Daemon [37].

## Results

### Questionnaires and Neuropsychological Measures

Repressors and sensitizers did not differ in mean age, verbal intelligence ^29^ or visual perception and organization ((WAIS-R, (*P*s>0.53)). Cronbach's α for the STAI state version was 0.86. Sensitizers were more anxious than repressors (*t* (38)  = −2.42, *P*<0.05). Cronbach's α for the STAI trait was 0.92. As expected, sensitizers had higher trait anxiety scores than repressors (*t* (38)  = −3.16, *P*<0.005). Cronbach's α for the BDI was 0.83. Sensitizers and repressors did not differ on the BDI (*P*>0.10) (see table 1).

### Behavioral Performance

We conducted t-tests for sum scores of evaluation ratings and latencies for all facial conditions (fearful, angry, happy, and neutral). Only ratings for the neutral face condition showed a significant difference between repressors and sensitizers (*t* (38)  = 2.10, *p* = 0.042), but not for latency (*t* (38)  = 1.69, *p* = 0.20). Repressors rated neutral faces as less threatening than sensitizers. Repressors tended to rate angry (*t* (38)  = 1.59, p = 0.12) and fearful (*t* (38)  = 1.46, p = 0.15) faces as less threatening than sensitizers, but these results, as for happy faces (*t* (38)  = 0.11, p = 0.92), did not reach statistical significance. In all conditions, sensitizers showed longer latencies than repressors, but differences were not significant (see table 2).

**Table 2:** Face evaluation task (fMRI experiment): evaluative ratings and latencies as a function of facial expression and coping style (low values indicate more perceived threat).

	Sensitizers		Repressors	
	*Mean*	*SD*	*Mean*	*SD*
Angry faces				
Rating	−0.9	0.3	−0.6	0.6
Latency	639.3	221.4	603.3	158.7
Happy faces				
Rating	1.3	0.3	1.3	0.4
Latency	583.8	205.3	526.3	143.3
Fearful faces				
Rating	−0.2	0.4	0.0	0.7
Latency	617.9	230.4	544.1	147.1
*Neutral faces*				
Rating	0.3	0.3	0.5	0.5
Latency	587.64	205.6	514.4	145.1

To examine the influence of coping on recognition memory, we carried out 2×2×4 ANOVAs with group as between-group independent variable and time of recall (immediate vs. delayed recall) and face condition (fearful, angry, happy, and neutral) as repeated measure independent variables. First, we analyzed total numbers of correctly remembered original faces (hits). There were main effects of time (*F* (1,38)  = 16.87, *p*<0.001) and emotion (*F* (3,36)  = 4.10, *p* = 0.013) for correctly recognized original faces. Subjects showed significantly more hits in the immediate than in the delayed recognition task and the most hits for the happy condition. No effects involving groups were found in the hit condition. Second, analysis of false positively identified distractors did not reveal a main effect for time or group, but a main effect for emotion was observed (*F* (3,36)  = 10.29, *p*<0.001). According to the results of paired *t*-tests, subjects produced more false alarms for happy and angry faces compared to neutral faces (p<0.05) (see table 3 for details). Additionally, we assessed participants' discrimination performance by calculating a discrimination index H-FA (hit rate (number of hits/30) – false alarm rate (number of false alarms/210)) (see table 4). The ANOVA results based on the discrimination index showed significant main effects of time (*F* (1,38)  = 18.88, *p*<0.001) and emotion (*F* (3,36)  = 3.82, *p* = 0.018). Participants manifested a better discrimination performance immediately after the fMRI experiment compared to the delayed recall condition and recognized happy faces better than neutral faces. No effects involving groups were observed for the discrimination index.

**Table 3:** Immediate (time 1) and delayed (time 2) recognition performance as a function of facial expression and coping style (number of correct recognitions (hits) and false alarms).

	Sensitizers		Repressors	
	*Mean*	*SD*	*Mean*	*SD*
**Hits**				
Angry time 1	15.9	5.9	14.9	6.1
Angry time 2	13.0	6.7	12.1	4.2
Happy time 1	16.7	6.6	16.0	6.4
Happy time 2	14.4	7.3	11.9	5.8
Fearful time 1	15.7	7.1	12.2	5.5
Fearful time 2	13.7	6.9	10.9	4.8
Neutral time 1	13.9	6.9	13.2	5.0
Neutral time 2	12.7	7.6	10.0	4.9
**False alarms**				
Angry time 1	7.7	6.8	7.4	4.8
Angry time 2	7.9	7.1	6.9	4.6
Happy time 1	7.7	7.7	7.3	6.2
Happy time 2	7.7	7.4	6.9	6.3
Fearful time 1	6.0	6.0	5.2	4.8
Fearful time 2	7.3	6.6	5.1	5.0
Neutral time 1	6.2	6.9	5.7	5.3
Neutral time 2	7.0	7.1	5.4	4.2

***Note: time 1 =  after 30 minutes; time 2 =  after 3 days.***

**Table 4:** Discrimination performance (H (hit rate (number of hits/30)) – FA (false alarm rate (number of false alarms/210))) as a function of facial expression and coping style.

	Sensitizers		Repressors	
	*Mean*	*SD*	*Mean*	*SD*
H-FA Angry time 1	0.49	0.17	0.46	0.19
H-FA Angry time 2	0.40	0.20	0.37	0.12
H-FA Happy time 1	0.52	0.20	0.50	0.20
H-FA Happy time 2	0.44	0.22	0.36	0.17
H-FA Fearful time 1	0.49	0.22	0.38	0.17
H-FA Fearful time 2	0.42	0.21	0.34	0.15
H-FA Neutral time 1	0.43	0.21	0.41	0.15
H-FA Neutral time 2	0.39	0.23	0.31	0.15

***Note: time 1 =  after 30 minutes; time 2 =  after 3 days.***

### Whole Brain FMRI Results: Between Group Comparison

#### Brain response to angry faces compared to neutral faces

**Figure 1:**
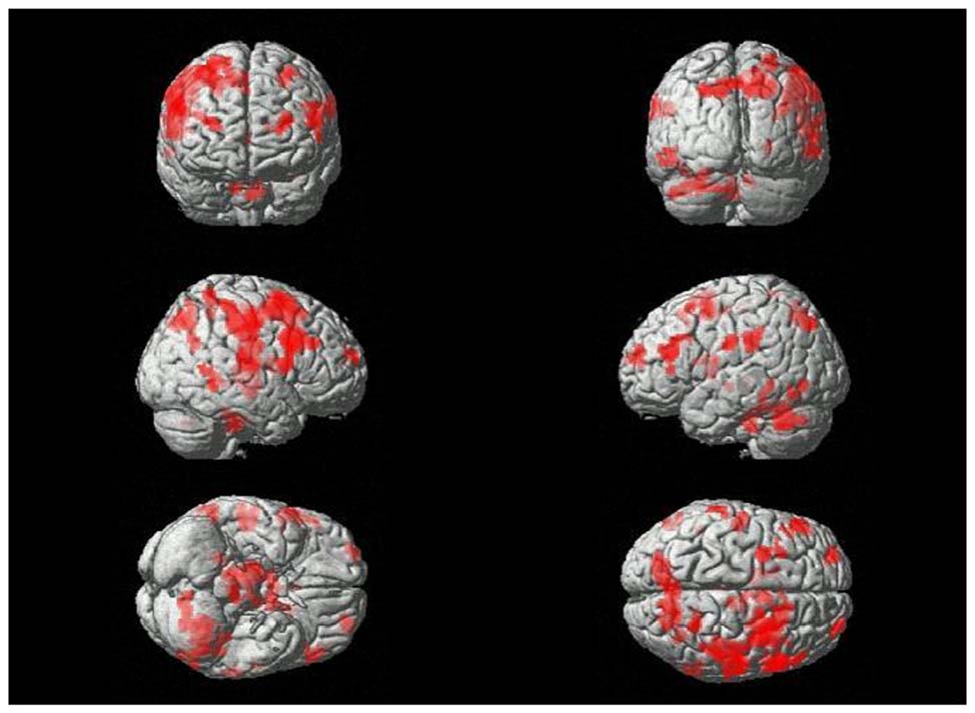
Between-group differences in brain response to angry facial expression compared to neutral faces. Enhanced brain activations of repressors compared to sensitizers (MNI coordinates). The activations are significant at p<0.05, k = 50 (FDR corrected). Reader's right is subjects' right.

Repressors showed greater activation in the superior, medial, middle and inferior frontal gyrus, superior temporal gyrus, precuneus, cerebellum, and inferior parietal lobe relative to sensitizing individuals. No significantly increased brain activation was found in sensitizing individuals. All data are significant at p<0.05, k = 50, False Discovery Rate corrected (FDR). Please see table 5 and Fig. 1 for details.

**Table 5:** Brain regions with heightened activation of repressors compared to sensitizers in the angry versus neutral face contrast.

Brain Region	BA^a^	Hemisphere^b^	MNI Coordinates			Size	Z-score^c^
			x	y	z		
Inferior frontal gyrus	9	R	60	12	28	633	5.06
Medial frontal gyrus	6	R	10	−22	56	153	3.99
Middle frontal gyrus	46	L	−50	32	20	213	3.98
Superior frontal gyrus	10	R	30	60	20	64	3.72
Superior temporal gyrus	39	R	36	−60	30	64	3.87
Inferior parietal lobule	40	L	−64	−30	28	97	3.61
Precuneus	7	L	−6	−70	48	760	3.94
Cerebellum	Declive	L	−30	−60	−30	534	3.96

a Brodmann areas.

b L = left, R = right.

c Coordinates of the maximal point of activation and the associated Z-values are shown. The activations are significant at p<0.05, k = 50 (FDR corrected).

#### Brain response to fearful faces compared to neutral faces

Repressive and sensitizing individuals showed no significant differences in brain activation (p<0.05, k = 50, FDR).

#### Brain response to happy faces compared to neutral faces

No significant differences were found in brain activation between repressive and sensitizing individuals (p<0.05, k = 50, FDR).

#### Brain response to neutral faces compared to angry faces

Sensitizers showed greater activation in the superior, medial, middle and inferior frontal gyrus, superior, middle and inferior temporal gyrus, fusiform gyrus, middle occipital gyrus, precentral gyrus, substantia nigra, thalamus, hippocampus, precuneus, cerebellum, and inferior parietal lobule relative to repressive individuals. No significantly increased brain activation was found in repressive individuals. All data are significant at p<0.05, k = 50, False Discovery Rate corrected (FDR). Please see table 6 for details.

**Table 6:** Brain regions with heightened activation of sensitizers compared to repressors in the neutral versus angry face contrast.

Brain Region	BA^a^	Hemisphere^b^	MNI Coordinates			Size	Z-score^c^
			x	y	z		
Inferior frontal gyrus	9	R	60	12	28	633	5.06
	44	R	54	6	12	633	4.44
	44	R	60	12	12	633	4.17
Medial frontal gyrus	6	R	10	−22	56	153	3.99
	32	R	4	6	52	153	3.52
	6	R	12	−14	54	153	3.11
Middle frontal gyrus	6	R	32	4	62	2334	4.48
	8	R	28	14	52	2334	3.96
	6	R	38	−2	54	2334	3.96
	6	L	−28	2	54	173	4.28
	6	L	−28	18	56	173	3.52
	46	L	−50	32	20	213	3.98
	46	L	−52	28	28	213	3.66
	9	L	−44	24	32	213	3.54
	10	L	−26	56	22	98	3.64
	10	R	30	60	20	64	3.72
Superior frontal gyrus	10	R	26	66	16	64	3.28
Superior temporal gyrus	39	R	36	−60	30	64	3.87
	22	R	62	−56	8	106	3.29
	22	R	58	−48	8	106	3.20
Middle temporal gyrus	22	R	58	−48	2	106	3.57
	22	L	−48	−44	−6	82	3.25
Fusiform gyrus	20	L	−38	−36	−22	82	3.19
	37	L	−40	−42	−16	82	3.18
Inferior temporal gyrus	19	L	−50	−70	−6	60	3.50
Middle Occipital gyrus	19	L	−58	−62	−12	60	3.41
Inferior parietal lobule	40	L	−64	−30	28	97	3.61
	40	L	−62	−40	40	97	2.90
Precuneus	7	L	−6	−70	−48	760	3.94
	7	R	2	−66	48	760	3.83
	7	R	20	−64	54	760	3.70
	7	R	26	−50	62	132	3.45
	7	R	26	−50	52	132	3.33
	7	R	16	−50	52	132	3.18
Precentral gyrus	4	L	−52	−16	30	123	4.04
Substantia nigra		R	16	−18	−10	942	4.73
Hippocampus		R	24	−42	−2	64	4.25
Thalamus	Ventr. Lat. Nc.	R	16	−14	14	942	4.21
	Gray matter	R	8	−22	−4	942	3.98
Cerebellum	Declive	L	−30	−60	−30	534	3.96
	Tuber	L	−48	−58	−36	534	3.83
	Declive	L	−26	−54	−22	534	3.73
	Culmen	L	−2	−32	−36	119	4.62
	Culmen	L	−2	−30	−24	119	3.44
	Culmen	R	10	−32	−32	104	3.80

a Brodmann areas.

b L = left, R = right.

c Coordinates of the maximal point of activation and the associated Z-values are shown. The activations are significant at p<0.05, k = 50 (FDR corrected).

#### Correlations between brain response to angry faces with memory for angry faces

To explore the relationship between activation in those brain areas where repressors manifested overactivation compared with sensitizers and recognition of angry faces we calculated for each participant mean activation scores averaged across all voxels for each of the eight clusters where group differences were observed. Then Product–moment correlations were calculated between mean cluster activation scores and memory for angry faces at time 1 and time 2 were calculated for sensitizers and repressors separately (see Table 7). Even though no significant correlations between brain response and recognition performance were found interesting differential correlation patterns were observed. Almost all correlations of brain activation with immediate recognition had a positive sign for both study groups. Sensitizers showed also exclusively positive correlations between brain response and delayed recognition but repressors manifested in four cases negative correlations with delayed recognition for angry faces.

**Table 7:** Correlations between mean cluster activation in regions with heightened response to angry faces (in repressors compared to sensitizers (see Table 5)) and discrimination performance H-FA for angry faces at time 1 and time 2 and loss of discrimination accuracy for angry faces from time 1 to time 2 (H-FA time1 minus H-FA time2) as a function of coping style.

Brain Region	Sensitizers H-FA			*Repressors H-FA*		
	time1	time2	time1-time2	time1	time2	time1-time2
Inferior frontal gyrus (xyz 60 12 28, k = 633)^a^	−.11	.01	−.18	.16	−.14	.31
Medial frontal gyrus (xyz 10 −22 56, k = 153)	.16	.22	−.12	.13	.11	.07
Middle frontal gyrus (xyz −50 32 20, k = 213)	.27	.34	−.17	.18	−.19	.37
Superior frontal gyrus (xyz 30 60 20, k = 64)	.21	.03	.24	.33	.09	.33
Superior temporal gyrus (xyz 36 −60 30, k = 64)	.00	.16	−.27	.11	−.06	.18
Inferior parietal lobule (xyz −64 −30 28, k = 97)	.10	.06	.05	.33	.07	.34
Precuneus (xyz −6 −70 48, k = 760)	.23	.15	.07	.03	−.14	.16
Cerebellum (xyz −30 −60 −30, k = 534)	.08	.11	−.06	.17	.17	.06

a Coordinates of the maximal point of activation within clusters, k =  cluster size.

Finally, an index for loss of discrimination accuracy over time was computed by subtracting discrimination accuracy at time 2 from discrimination accuracy at time 1 (H-FA time 1 – H-FA time 2). Sensitizers showed five negative correlations between brain response to angry faces and loss of discrimination accuracy over time. This means that higher activation in response to angry faces at encoding tended to be associated with less memory loss for angry faces over time. Repressors manifested instead exclusively positive correlations between brain response to angry faces and loss of discrimination accuracy. Thus, for repressors higher activation in response to angry faces at encoding tended to be related to more memory loss for angry faces. To compare strength of correlation of brain response with memory loss between groups Fisher's Z was applied. For the correlation between middle frontal gyrus activation and memory loss a significant difference between groups was obtained ((*z* = 1.64, *p* = 0.05, one-tailed). For the correlations of inferior frontal gyrus and superior temporal gyrus activation with memory loss marginally significant differences between groups in height of correlation were observed ((*z* = 1.47 and *z* = 1.34 *p*<0.10, one-tailed).

## Discussion

The aim of this study was to test the discontinuity hypothesis using fMRI. We investigated the influence of coping style on brain activation during memory encoding of emotional faces and the relationship between coping style and the recall of threatening and non-threatening faces. To date, the discontinuity hypothesis has been investigated by behavioral studies only.

Subjects evaluated emotional faces during the fMRI session. We found activation differences between repressors and sensitizers that confirm in part our hypothesis. Repression was associated with increased cortical activation in frontal, parietal, temporal, and occipital areas and the cerebellum during encoding of directly threatening faces (i.e. angry facial expression) but not as hypothesized during encoding of indirectly threatening faces (i.e. fearful facial expression). As expected, the happy versus neutral contrast did not show any significant activation differences between repressors and sensitizers. Sensitizers did not exhibit heightened neural activation in any of the contrasts compared to repressors. The present anger-specific findings may be due to the directly threatening character of angry facial expression (with direct eye gaze - as used in our experiment) compared to fearful facial expression. Angry facial expression signals a potential impending attack (of the person opposite the observer). Fear faces are not signalling direct threat to the observer but indicate the existence of a threatening event or stimulus in the environment [38]. Therefore, fearful faces can be interpreted as signals requiring clarification regarding the spatial source of a potential danger and therefore seem not directly threatening. Our evaluation data are also in line with this interpretation because fearful faces were rated as less threatening than angry faces (see table 2). According to the repressive discontinuity hypothesis [39] and the vigilance-avoidance theory [40] repressors are very sensitive to self-relevant threats at the first stage of processing. On this background it appears plausible that repressors show high reactivity to directly but not to indirectly threatening faces.

Sensitizers exhibited heightened neural activation only in the neutral versus angry contrast compared to repressors: sensitizers' activation was increased in the superior, medial, middle and inferior frontal gyrus, superior, middle and inferior temporal gyrus, fusiform gyrus, middle occipital gyrus, precentral gyrus, substantia nigra, thalamus, hippocampus, precuneus, cerebellum, and inferior parietal lobule relative to repressive individuals. No significantly increased brain activation was found in repressive individuals for this contrast. Taken together, sensitizers show increased activation in brain regions which are important for attention control and appraisal. A neutral face may represent a potential threatening emotion expression because it can be perceived as unclear and ambiguous. This may lead sensitizers to process neutral faces more intensively than clearly interpretable angry faces. In contrast to repressors, sensitizers show high reactivity to potentially or covertly but not to directly threatening faces. In particular, neutral faces can signal uncertainty or mask an emotional expression whereas angry faces clearly express threat and are less ambiguous.

The behavioral data revealed no main or interaction effect of coping style for immediate and delayed memory. There was a main effect of time for recognition performance. As could be expected, memory recognition was much better during immediate testing than it was 3 days later. In the evaluation task, no significant differences between repressors and sensitizers were found for threatening (i.e. angry and fearful) or happy faces. Only ratings of neutral faces were significantly influenced by coping style. Repressors tended to rate neutral faces more positively than sensitizers, as well as angry, happy, and fearful faces. But, these results did not reach statistical significance. Thus, our hypothesis that repressors evaluate emotional faces as less threatening than sensitizers was not confirmed by our data. No significant differences between repressors and sensitizers were observed for evaluation latencies.

The ability to recognize complex emotion in facial expressions is a critical evolutionary difference between humans and other species. Facial expression serves as an important factor in daily communication and influences brain activity in the successful encoding of faces. This ability is especially important for threatening faces because it improves chances of survival [19]. Since we were interested in the emotional impact of threatening (and non-threatening) facial expressions on brain activation during encoding, we investigated the differential emotional effects by comparing neural response to emotional faces with that to neutral faces.

The model of coping modes [25] describes habitual behavior tendencies in anxiety-provoking and threatening situations. Referring to this model, repression is characterized by cognitive avoidance of threatening stimuli to shield the organism from distressing stimuli. Repressors try to prevent an uncontrollable increase in arousal. According to the repressive discontinuity hypothesis, repressors manifest a discontinuity in memory processing of threatening information. Repressors were found to be sensitive to threat in early, perceptually driven phases of information processing (i.e. encoding), whereas they manifested weak memory representation of potentially threatening stimuli during memory retrieval [9], [10]. Previous behavioral studies did not investigate neural processes and emotional face expressions. In this novel fMRI study, repressors differed from sensitizers on a cortical processing level during the encoding of directly threatening faces but not during the encoding of indirectly threatening (i.e. fearful) and non-threatening or positive (i.e. happy) faces. Thus, we observed a differential increase of neural response to angry faces in repression compared to sensitization. Contrary to expectations, fearful facial expression did not cause stronger cortical response in repressors than sensitizers. This finding appears to be in line with the idea that repressors are not especially sensitive to not self-relevant threats [39]. We found the most remarkable activation differences in the DLPFC (BA 9, BA 10 and BA 46) for the angry versus neutral contrast. Other regions with heightened activation included BA 6, the superior temporal gyrus (BA 39), inferior parietal lobule (BA 40), precuneus (BA 7) and cerebellum (declive). This heightened activation during the encoding of angry faces may demonstrate an enhanced initial processing of threatening information among repressors supporting the discontinuity hypothesis, i.e. repressors do not withdraw their attention from threatening stimuli at early stages of emotion processing but they manifest a perceptual hypersensitivity for direct threat.

However, contrary to our hypothesis we found no evidence that repressors' memory for threatening faces was better directly after encoding and worse after three days compared to that of sensitizers. Immediate and delayed recall performance for angry facial expression did not differ between study groups. Interestingly, our findings of increased activation of the middle and medial frontal cortex (BAs 46, 6) as well as the inferior frontal cortex and the superior temporal gyrus during evaluation of angry faces can be interpreted as enhanced encoding of this directly threatening facial expression. Activation in frontal areas (BA 46 and BA 6) during encoding has been found previously to predict recognition of facial expression [41]. Moreover, Jackson et al.'s fMRI findings [42] showed that superior temporal and inferior frontal areas are specifically recruited in the service of visual short-term memory for angry faces. It has been repeatedly shown that the prefrontal cortex plays an important role in the memory encoding of faces and emotional stimuli [43], [44] as well as for cognitive reappraisal and emotion regulation [45]. Especially, the DLPFC, inferior frontal gyrus, inferior parietal lobule seem to be crucial for the successful encoding of faces [19]. In the past, the cerebellum was not regarded as playing an important role during emotion processing, but a recent memory recognition study [18] found cerebellar activity for negative faces which is complementary to our results of heightened activation in the cerebellum for angry faces.

Thus, in view of the latter results from neuroimaging research it can be assumed that more activation of fronto-temporal areas during the processing of angry faces in repression compared to sensitization indicates more initial encoding of directly threatening stimuli. Remarkably, the enhanced response to anger faces in memory-relevant areas did not lead to better recognition of these faces neither immediately after encoding nor after a delay of three days. In this perspective, a repressive discontinuity between neurobiological encoding and (immediate as well as delayed) retrieval of threatening information could be established. Even though we could not corroborate the prediction of the discontinuity hypothesis according to which repressors should exhibit poor delayed memory for threatening stimuli compared to individuals with other coping styles we found evidence for a discrepancy between extent of neural activation in encoding-relevant brain areas in response to anger faces and subsequent memory for these faces. It is possible that heightened right DLPFC response in repression might indicate inhibition of threat processing. Increased activation in prefrontal areas may be associated with attentional control over threat-relevant stimuli. Bishop et al. [46] showed increased recruitment of the DLPFC in low anxious subjects during presentation of threatening stimuli and suggested heightened top-down control mechanisms. In our study, repressors described themselves as low-anxious. Therefore, our findings may also reflect heightened attentional and top-down control in repression during the encoding of threatening faces. Inhibition of threat processing could explain why despite of enhanced activation in encoding-relevant areas in repressors compared to sensitizers no differences in memory performance were observed.

Our results support the repressive discontinuity hypothesis on a neurobiological level in the sense that repressive individuals show stronger brain reactions to self-relevant threats than sensitizing individuals [39], [40], [47]. It has been assumed that repressors exhibit an initial vigilant stage during quick and early emotion processing which is followed by an avoidant stage with more controlled strategies. Our neuroimaging results may reflect this enhanced sensitivity towards angry faces during encoding. Schwerdtfeger et al. [48] reported that repression is associated with initial rapid engagement, but is then followed by disengagement from threatening faces. They found significant differences for angry faces, but not for happy or neutral faces during a spatial cueing task. Those results were similar to ours as we only found significant group differences for the angry condition. Furthermore, our results are in line with one of our previous studies [23] which showed that repressors exhibit stronger neural responses in cortical areas of emotion processing during presentation of masked threatening faces. Paul et al. [23] investigated the initial step of emotion perception (automatic emotion processing) whereas this study examined explicit encoding of emotions (i.e. controlled emotion processing). Interestingly, analogous to our present findings Paul et al. described also stronger responses in cortical areas of emotion processing during presentation of masked threatening faces in repression but no differences in detecting masked faces. Thus, stronger neural response to threatening information seems not to be associated to better cognitive performance (more efficient stimulus detection or memory) as could be expected. These findings could be due to cognitive avoidance of threatening stimuli in repressors at quite an early processing stage in order to shield the organism from further processing of the distressing stimuli. Since in the present experiment repressors showed enhanced brain activation during the explicit evaluation and encoding of threatening faces it appears that the initial vigilance response of repressors to threat triggering physiological responses is not necessarily limited to automatic or non-conscious processes but can also include explicit threat processing.

In a recent electrophysiological study measuring event-related brain potentials an enhanced responsivity to angry faces in prefrontal areas and more right-lateralized activation were found in repressors [49]. These findings are in line with our results of heightened activation of repressors in the right BA 9 during the encoding of angry faces. Furthermore, our neuroimaging results are consistent with the study of Sander et al. [11] which revealed heightened prefrontal activation as a main effect and an increased activation in temporoparietal regions in repression during perception of sad stimuli.

As mentioned above, our memory results do not support the discontinuity hypothesis. Krohne and Hock [10] and Hock and Krohne [9] showed that repressors have a good memory for aversive pictures and ambiguous sentences shortly after encoding, but poor memory for aversive pictures and potentially threatening sentences during delayed memory testing after 3 days. The absence of repressive memory effects in our study may originate from a rather low number of participants. Both samples in the above-mentioned behavioral studies included substantially more participants than our sample. Furthermore, we used faces only as stimuli whereas Krohne and Hock [10] administered faces, bodies, and the interaction between people as stimulus material.

In a directed forgetting study, Myers et al. [50] found that repressors had poor recall for negative stimulus material. They forgot more negatively valenced words than non-repressors when instructed to forget those previously shown words. There were no significant differences if subjects were instructed to remember words and no significant differences for positively valenced words. Myer et al.'s findings suggest that repressors have an enhanced capability for using retrieval inhibition. Furthermore, it was shown that repressors forgot negative words only if those words were self-relevant and under private circumstances [51]. One possible reason we did not find any significant differences in the memory tasks is because subjects were instructed to remember emotional faces. In addition, our facial stimuli may not be self-relevant enough and the study design not perceived as private enough to reveal differences between repressors and sensitizers. But, another study found that repressors exhibited lower free recall of negative, self-relevant stimuli during an explicit memory task, but their implicit memory for the same information was not affected [52]. Taken together, other stimuli, such as emotional scenes and sentences, might match a subject's individual memory in a more general way and would therefore be more likely to be interpreted by repressors as self-relevant negative information.

The setting of our study may have led to overriding the habitual avoidance response among repressors in favor of self-presentational goals [53]. This could be another explanation why we did not find any differences in behavioral responses. Furthermore, Cutler et al. [54] suggested that repressors deny unpleasant emotional experiences during encoding, while only moderately biasing the recognition and recall of unpleasant affect.

It can be criticized that we administered an intentional or explicit learning task in our study to test the repressive discontinuity hypothesis. It has been found that when repressors are instructed to remember stimuli the impaired delayed recognition performance for threat is removed or reversed [50], [55]. In this regard, it has been argued that repressors might be more motivated than sensitizers to remember stimuli in intentional learning tasks. When looking at our recognition data there is no evidence that repressors were better in recognizing facial expressions immediately after the fMRI experiment (but also after three days). Taking into consideration the fMRI data showing a specific hyper-activation of encoding- relevant brain areas in response to angry faces in repressors it appears also unlikely that repressors were in general more motivated or engaged to remember stimuli than sensitizers. However, we cannot rule out that our instruction to memorize faces counteracted repressors' tendencies of cognitive avoidance leading to poor delayed recognition of threat. We decided to give the explicit instruction to memorize faces because we wanted to examine neural correlates of encoding processes as a function of coping style. Future research on the repressive discontinuity hypothesis should administer covert or implicit memory tasks (e.g., gender decision) so that implicit memory processes can be investigated as a function of repressive coping style. Such findings could complement the present results on the explicit encoding of threatening faces in repression.

We did not find any main or interaction effects of coping style on recognition performance. Interestingly, for number of hits (correctly remembered facial expression) we obtained main effects of time and emotion. As expected, memory was better for immediate than delayed recall. Moreover, subjects exhibited more hits for happy faces. Shimamura et al. [56] reported better memory for happy facial expressions than angry, fearful and surprised facial expressions which is consistent with our findings. Those authors argued that the smile of a happy face directs attention toward the particular person and could therefore be remembered better.

The face evaluation task revealed a significant difference between repressors and sensitizers for neutral faces. Sensitizers rated neutral faces as more threatening than repressors. Neutral faces can be perceived as ambiguous, because the expression is neither emotionally positive nor negative. Therefore, it could be perceived as potentially threatening [57]. Sensitizers' inability to tolerate uncertainty may lead them to regard neutral faces as more threatening.

### Limitations

Several limitations should be acknowledged in the current study. The number of subjects was relatively small. We studied 40 subjects who were recruited from a total sample of 150 females. Additionally, future neuroimaging research should use threatening sentences and pictorial stimuli [9], [10]. We included only women to avoid gender bias because Krohne et al. [10] observed gender differences when testing the discontinuity hypothesis. Another study also found gender differences during the encoding of faces [58]. Future imaging research should address this issue and should also include information about the female hormonal cycle because the neural response to negative emotional stimuli was modulated by sex hormones and the female hormonal cycle [59]–[61]. As mentioned above, the administration of an explicit learning task could be a limitation of the present study. Implicit learning tasks seem to be more appropriate to reveal repressive inhibition effects in delayed memory for threatening information [10], [62].

## Conclusions

Our findings suggest that repressive coping style influences the initial neural processing of emotional information in cortical areas relevant for encoding and emotion regulation, which confirms the assumption of hyper-responsivity to directly threatening information in repression at an early processing stage. We observed a discrepancy between high neural activation in encoding-relevant brain areas in response to angry faces in repressors and no advantage in subsequent memory for these faces compared to sensitizers. Coping resources play a major role in the development and maintenance of stress related mental disorders [2]. Therefore the investigation of coping styles in mental disorders should be addressed in future neuroimaging studies to improve our knowledge about the etiology, treatment and prognosis of mental disorders, such as depression, anxiety-related disorders, and posttraumatic stress disorders.
